# Representation of Rural Older Adults in AI for Health Research: Systematic Literature Review

**DOI:** 10.2196/70057

**Published:** 2025-09-15

**Authors:** Kristina Shiroma, Jacqueline Miller

**Affiliations:** 1 School of Information Studies, Louisiana State University, 223T Peabody Hall, Baton Rouge, LA, 70803, United States, 1 2255787932

**Keywords:** artificial intelligence, aging, older adult, rural, health care research, Preferred Reporting Items for Systematic Reviews and Meta-Analyses, PRISMA, systematic literature review

## Abstract

**Background:**

Older adults in rural communities face unique challenges, including geographic isolation, a shortage of health care providers, and limited access to specialized services. Artificial intelligence (AI) has emerged as a promising solution for improving health care access and delivery. However, concerns persist about equitable access and representation in these innovations, especially for marginalized populations where technological literacy and infrastructure may present additional barriers to effective use.

**Objective:**

This systematic literature review aims to: (1) identify existing literature on AI for health research focused on rural older adults, and (2) critically evaluate the identified literature to highlight gaps and inform the development of inclusive AI for health research and design practices.

**Methods:**

Between January 2024 and March 2025, we followed the PRISMA (Preferred Reporting Items for Systematic Reviews and Meta-Analyses) 2020 Protocol to conduct a systematic search of health and computer science literature. We searched 7 databases (PubMed, CINAHL Plus with Full Text, PsycINFO, Web of Science, IEEE Explore, ACM Digital Library, and Scopus) to identify relevant research papers published between January 2013 and December 2023. We used predetermined search terms and built-in result limiters, including English language, human subjects, full text, and aged 65+. Publications were excluded if they were not empirical or did not include a focus on older adults, rural populations, or AI. The resulting data were reviewed, coded, and analyzed using thematic analysis.

**Results:**

A total of 23 papers comprised the final sample. The results showed that the representation of rural older adults in AI for health literature is limited. We identified three salient themes: (1) Numbers over Narratives: The Quantitative Focus in AI for Health Research on Older Adults, (2) Efficacy over Impact: Prevalence Clinical Outcomes in AI for Health Research, and (3) Deepening Disparities: Representation of Rurality Missing in AI for Health Research. These themes underscore the need for a more nuanced understanding of how AI for health research can be tailored to the specific needs of rural older populations.

**Conclusions:**

Our systematic analysis identified a robust body of research on AI for older adults. However, a critical gap emerged with a dearth of studies explicitly focusing on older adults in rural communities. This lack of representation raises concerns about the generalizability of findings and the potential for exacerbating existing health care disparities in rural areas. Future research should: (1) prioritize targeted recruitment strategies for rural older adult participants to ensure better representation in AI for health research; (2) develop community-based AI policies, practices, and products that reflect the specific needs and contexts of rural populations; and (3) explore solutions that address the limited representation of rural communities, ensuring that AI interventions are equitable, accessible, and beneficial for all.

## Introduction

### Background

The global aging population presents significant opportunities to expand and extend health care, and artificial intelligence (AI) offers promising avenues to address these issues. As the population gets older, successful AI integration into health care systems necessitates a deeper understanding of the specific needs and perspectives of older adults. The increasing prevalence of chronic health conditions among older adults intensifies the demand for health care services while also offering new opportunities for engagement through digital health tools [1]. AI holds promise for meeting these needs, from early disease detection [2] to personalized care management [3] and even robotic assistance for independent living [4]. However, the integration of AI into health care is not without significant challenges. To be effective, AI-powered health care tools must be user-friendly, accessible to individuals with varying levels of technological literacy, and free from algorithmic biases that could disproportionately affect older populations [5].

### Rurality and Older Adults’ Health

The demographic shift toward an aging population is particularly pronounced in rural areas, where access to health care is often limited. Over the past decade, 106 rural hospitals in the United States have closed [6,7], and another 600 are at risk of following suit [8], leaving rural communities increasingly vulnerable to limited access to essential health care services. These hospitals have traditionally provided vital care to large populations of older adults, but as closures continue, there presents a need to rethink traditional in-person health care models. Approximately 20% of the US population, or 60 million people, live in rural areas, and they face disproportionately worse health outcomes compared to urban populations [9]. Rural older adults, in particular, experience higher rates of comorbidities, such as obesity, cardiovascular disease, and stroke, as well as a growing number of emergency department visits [9,10]. This has contributed to widening life expectancy gaps between rural and urban populations [11]. In addition, rural communities face unique barriers that make it harder for older adults to access the specialized care required to address these complex health challenges [10]. Although online care models and other AI for health solutions are beginning to demonstrate promise in improving access to specialized health care, many rural areas still lack the necessary infrastructure to implement these solutions [12]. Ensuring AI health care solutions address these challenges will be crucial in providing equitable care for rural older adults.

### AI for Health Research

We use the phrase “AI for health research” to refer to the investigation and development of AI technologies to improve various aspects of health care. By leveraging machine learning, natural language processing, and other AI techniques, researchers aim to create systems that can process vast amounts of health data to enhance clinical decision-making as well as to deliver highly personalized and sometimes remote care. However, despite its potential, AI for health research faces several significant challenges [13]. One of the most pressing issues is the quality and representativeness of the data used to train AI systems [14]. Large datasets are essential for AI models; however, many of these datasets may be incomplete, biased, or not sufficiently diverse, raising concerns about the accuracy and fairness of AI-generated decisions [2]. Furthermore, the sensitive nature of health data raises ethical concerns, particularly around privacy, security, and the risk of misuse. Ensuring robust data protection mechanisms is crucial to maintaining public trust in AI-driven health care solutions [15].

Another major challenge lies in the “black box” nature of many AI algorithms, which makes it difficult for health care providers and patients to understand how decisions are made [16]. This lack of transparency undermines accountability and raises the potential for unrecognized biases and amplified health care disparities [17,18]. Research continues to show that top-down or prescriptive design processes in the development of technology, including AI, often fail to include the perspectives of older adults, overlooking their unique experiences within their communities [19]. However, when leveraging the knowledge of this group through community-based research practices, technology design can be more effectively tailored to address issues, such as usability, literacy, privacy, and data management [19]. In addition, there is a significant gap in understanding the perspectives of older adults and other marginalized populations, such as those in rural areas. Without incorporating their diverse experiences and needs, AI tools risk failing to address the specific challenges faced by these groups, potentially exacerbating existing health inequities [20]. Addressing these challenges is essential to ensuring that AI for health research leads to equitable, effective, and ethically sound health care solutions.

### Study Aims

Building on the insights from the existing literature, it is clear that gaps remain in understanding how AI for health research can effectively address and incorporate the health care needs of rural older adults. This systematic literature review aims to: (1) identify existing literature on AI for health research specifically focused on older adults residing in rural communities, (2) critically evaluate the identified literature to highlight gaps, and (3) inform the development of inclusive AI for health research and design practices.

## Methods

### Study Design

We followed the PRISMA (Preferred Reporting Items for Systematic Reviews and Meta-Analyses) 2020 protocol for systematic literature reviews [21]. The PRISMA 2020 checklist is provided as Checklist 1. A protocol was not registered for this systematic literature review. A systematic review methodology was selected to enable a focused, critical appraisal of how rural older adults are represented in empirical AI for health research. Given the equity implications of AI in health care, a systematic review was appropriate to assess not only the presence but also the depth and nature of rural older adult representation using predefined inclusion criteria, structured data extraction, and formal critical appraisal tools (eg, Joanna Briggs Institute [JBI]).

We performed 4 rounds of systematic selection and review for the 7 selected databases (PubMed, CINAHL Plus with Full Text, PsycINFO, Web of Science, IEEE Explore, ACM Digital Library, and Scopus). In Round 1, we searched the databases using predetermined keywords. In Round 2, we screened titles and abstracts using predetermined inclusion and exclusion criteria. In Round 3, we further screened the full text of the selected papers using the same predetermined inclusion and exclusion criteria. Finally, in Round 4 we reviewed and coded each study in the final sample. A detailed description of our search strategies can be found in Multimedia Appendix 1. Once the articles were coded, the researchers met to analyze the data using thematic analysis [22]. The main themes identified centered on the representation of older adults in AI for health research.

In addition, we assessed the levels of evidence reported in the 23 papers [23-45] using the appraisal tools developed by the JBI to evaluate study quality [46]. To calculate assessment scores, 1 point was assigned to each question answered as “Yes,” while responses of “No” or “Unclear” received 0 points. The total score was then divided by the number of questions and multiplied by 100 to yield a percentage. Based on the resulting score, studies were categorized as very poor (0%‐30%), poor (31%‐50%), fair (51%‐70%), good (71%‐90%), or excellent (>90%). Both authors reviewed and independently completed the checklist for each study; disagreements were resolved through discussion.

### Ethical Considerations

This systematic literature review was deemed exempt by the Louisiana State University Institutional Review Board.

## Results

### Search and Screening Outcomes

Round 1, keyword search, yielded 1112 publications across all databases, with 479 publications from medical databases and 633 from general and computing databases. After initial screening for the predetermined exclusion criteria, 303 remained for title and abstract screening in Round 2. During Round 2, a total of 278 publications were removed based on the predetermined exclusion criteria, leaving 25 publications for screening in Round 3. During Round 3, full-text screening, 2 publications were excluded.

**Figure 1.**
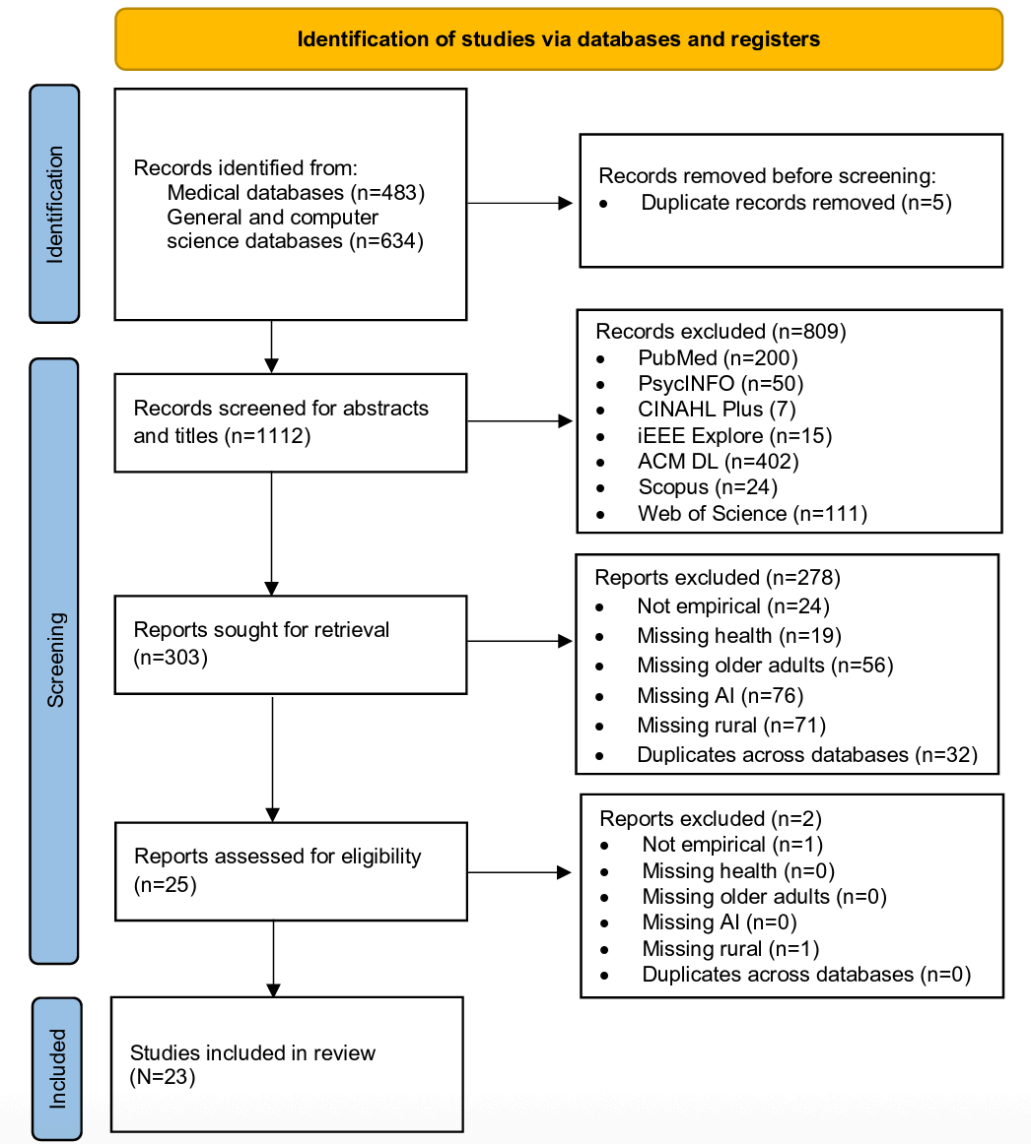
Preferred Reporting Items for Systematic Reviews and Meta-Analyses (PRISMA) flow diagram. AI: artificial intelligence.

This round resulted in a final sample comprised of 23 publications [23-45]. Among these, 8 publications [23-30] selected from the medical databases (PubMed, CINAHL Plus with Full Text, and PsycINFO) and 15 publications [31-45] selected from the general and computer science databases (Web of Science, Scopus, ACM DL, and IEEE Explore). The final PRISMA search strategy with results is illustrated in Figure 1 below.

### Quality Assessment Outcomes

To evaluate the methodological quality of the included studies, we used the JBI Critical Appraisal Checklists. Each study was independently reviewed by 2 researchers, with discrepancies resolved through discussion to ensure consistency and minimize potential bias. Studies that met the majority of JBI criteria were retained for inclusion in the final synthesis, ensuring a high-quality evidence base. Results of the quality appraisal are provided in Multimedia Appendix 2. Of the included studies, 10/23 (43%) [23,25-27,36-39,43,45] were rated as excellent, 10/23 (43%) [24,28,29,31-34,40,41,44] as good, and 3/23 (13%) [30,35,42] as fair in overall methodological quality.

### Descriptive **Statistics**

A summary of the characteristics of all 23 studies [23-45] is presented in MultimediaAppendices 3  4. Of the 23 [23-45] publications, the years of publication ranged from 2014 to 2023; the majority of the research has been published since 2021 (16, 70%) [23-27,29,33-38,42-45]. Studies were conducted in Asia (13, 57%) [23-27,29,33-35,39-41,45], Europe (2, 9%) [30,43], North America (7, 30%) [28,31,32,36,38,42,44], and Africa (1, 4%) [37]. Fields of study represented in this sample included health sciences (15, 65%) [23,26,30,32,33,35-40,42-45], psychology (3, 13%) [24,25,31], neuroscience (2, 9%) [29,34], gerontology (2, 9%) [27,41], and rehabilitation (1, 4%) [28].

Quantitative methods were almost exclusively used (19, 83%) [23-27,29,30,34-45]. The quantitative studies focused on aspects of AI in health research, such as prediction [24-26,42], screening [23,37], investigating [27,43], estimating [29], evaluating [30,38-41,44], and design and development [34-36,45]. The qualitative studies (4, 17%) [28,31-33] focused on patient experiences of robotic stroke therapy [28], the demand for smart health care services for older adults [33], and participatory design of socially assistive robots for mental health [31,32].

Sample sizes across the included studies ranged from as few as 10 to over 1.85 million older adult participants. Definitions of what constituted an “older adult” varied considerably. Participant ages spanned from 50 to 105 years, reflecting a lack of consensus on age-based eligibility criteria. Two studies did not specify age-based criteria but noted that participants were recruited from older care facilities, implying an assumed classification based on setting rather than age. It is important to note that there is no universally accepted standard for defining “older adult.” The World Health Organization generally defines older adults as individuals aged 60 years and older, particularly in global and low- to middle-income contexts [47]. In contrast, the United States Census Bureau designates age 65 years as the threshold for older adulthood [48]. These variations highlight how classifications of “older adult” may differ by region, cultural context, or institutional policy.

The representation of rurality similarly varied across the studies. Only 7/23 (30%) [23,26,28,30,35,41,44] of the publications included in this review focused entirely on a rural population. The context of rurality in these studies included rural villages in Yangxi County, China [23]; villages and towns in rural districts of Beijing [35]; home-based care in rural China [41]; rural areas in the state of Georgia, United States [28]; small rural hospitals in the United States [44]; rural regions of South Korea [26]; and a rural health care center in Sweden [30]. However, rurality was often (14/23, 57%) [24,25,29,31-34,36-38,40,42,43,45] treated as a background variable used for comparison against urban settings rather than as a distinct sociocultural or structural context worthy of focused analysis. Furthermore, nearly half of the studies (11/23, 49%) [23-25,27,33-35,39-41,45] examining AI for health in rural areas were conducted in China, suggesting a potential geographic bias or overrepresentation that may limit the broader applicability of findings and obscure rural-specific insights from other regions.

The types of AI used across the included studies were primarily described as machine learning (n=7, 30%), which supported a range of predictive tasks, including identifying depression risk [24], forecasting cognitive decline [25], modeling active aging [27], predicting suicidal ideation in rural older adults [26], and developing risk scores for stroke and coronary heart disease [34,36,45]. Deep learning (n=1, 4%) was used for image-based screening, specifically for detecting retinal lesions in rural older adult populations [23]. Robotics (n=2, 9%) was applied in studies involving telerehabilitation for rural veterans [28] and remote consultation through robot-assisted echocardiography [30]. One additional study [31] described the ongoing participatory design of socially assistive robots with older adults, though it had not yet reported implementation outcomes. Notably, no studies examined the use of generative AI (eg, large language models). Moreover, few studies included qualitative data capturing older adults’ own perceptions, experiences, or understanding of AI in their health care, limiting insight into how this population interprets, evaluates, or engages with AI technologies in practice.

AI was primarily examined as a strategy to extend health care services to remote and underserved areas. The largest proportion of studies (10/23, 43%) [23-26,31,34,36,37,42,45] focused on AI as a screening and predictive tool in rural health care settings, supporting early identification of retinal lesions [23], depression [24,31], cognitive decline [25], suicidal ideation [26], stroke risk [34], early hospitalizations due to ambulatory-care sensitive conditions [36], diabetes [37], novel disease association [42], and cardiovascular disease [45]. Fewer studies explored other applications of AI, including its use in robotic telerehabilitation, particularly for rural veterans with limited access to in-person care [28]; its role in supporting long-term public planning and social support through epidemiological modeling in aging populations [29]; and its utility in facilitating remote specialist consultation, such as through robot-assisted echocardiography that reduced time to care [30]. These findings highlight a dominant focus on AI’s predictive capabilities in rural contexts, while pointing to emerging but underexplored applications in rehabilitation, planning, and remote access.

## Discussion

### Principal Findings

Across the literature, we identified three salient themes: (1) Numbers over Narrative: The Quantitative Focus in AI for Health Research on Older Adults, (2) Efficacy over Impact: Prevalence of Clinical Outcomes in AI for Health Research, and (3) Deepening Disparities: Rural Older Adults Missing in AI for Health Research. We examine these themes in the following sections.

#### Theme 1: Numbers Over Narratives: The Quantitative Focus in AI for Health Research on Older Adults

Across this systematic literature review, we identified that AI for health research predominantly emphasizes quantitative findings, focusing on measurable results, such as diagnostic accuracy, predictive modeling, and algorithmic efficacy. This trend reflects broader scientific priorities placed on numerical data and technical performance, often at the expense of deeper, qualitative insights into the lived experiences of older adults.

The studies reviewed generally included older adults as subjects of analysis, rather than as active participants in the research process. This suggests, and in many cases perpetuates, a research approach that focuses on older adults, rather than with them. By emphasizing AI’s reach and capabilities in health research, such as screening [23,37], prediction [24-26,40,42], identification [27,38,43], and feasibility [30], the literature largely overlooks the personal narratives and lived experiences that are crucial for designing patient-centered, effective AI solutions. For example, Cui et al [23] designed their quantitative study to test the efficacy of AI as a screening tool for rural older adults who are particularly more susceptible to retinal lesions. This application of AI, like most of the studies in this review, is provider-focused, as older adults were not asked to contribute their perspectives on the use of AI within their health care.

This focus on quantitative findings, while valuable in establishing initial data and baseline efficacy, remains a limitation in fully understanding the potential and impact of AI for health technologies [5]. The minimal presence of qualitative research undermines the broader relevance and applicability of AI solutions for marginalized populations, particularly rural older adults. Among the few studies that incorporated qualitative components, many offered limited depth or treated qualitative insights as secondary. For example, Lee et al [33] conducted a qualitative study with older adults in China and found that 81.6% of participants had never independently purchased or leased smart health care products, highlighting low levels of engagement and understanding of these tools, despite their increasing availability. Studies focused on clinical outcomes and algorithmic performance often fail to address the real-world implications of these technologies, such as usability, trust, and accessibility, which present critical factors in adoption and long-term success, especially in rural communities.

Most qualitative studies explore the experiences, preferences, and barriers faced by older adults [19]. This approach not only enriches the findings of quantitative studies but also ensures that AI for health tools is designed in ways that truly meet the needs of older adults, especially those in underrepresented communities. Without this qualitative dimension, the development of AI technologies will likely continue to miss crucial elements that could optimize their utility in everyday health care settings, particularly in rural and underserved populations. The current literature gap underscores the need for a more balanced research approach that integrates both quantitative and qualitative methods, ensuring that emerging technologies are both scientifically rigorous and human-centered.

#### Theme 2: Efficacy Over Impact: Prevalence of Clinical Outcomes in AI for Health Research

Our systematic literature review highlighted the prevalence of clinical outcomes in AI for health research within the context of rural older adult populations. A majority of studies in this sample concentrated on the efficacy of AI interventions designed to evaluate existing models or outcomes (6/23, 26%) [28,30,37-39,44], predicting health outcomes (4/23, 17%) [24-26,42], investigating determinants of health (3/23, 13%) [27,33,38], screening medical data (2/23, 9%) [23,45], estimating disease prevalence (1/23, 4%) [29], and analyzing associated factors (1/23, 4%) [41]. While the investigations of these outcomes are pivotal and promising, our review suggests that a more inclusive, participatory approach may ensure that AI tools are better aligned with the challenges and needs of rural older adults. In contrast to the clinical-centric focus, other technology sectors have increasingly adopted community-based participatory research models to engage users in the design and development process [20]. However, such approaches have yet to be widely applied in AI for health research, as seen in this systematic literature review.

Our findings suggest that while AI-driven tools demonstrate potential in improving diagnostic precision and personalizing care for older adults [2-4], the understanding of their real-world application in rural areas remains underexplored. Rural health care systems are complex, with challenges, such as limited broadband access, shortages of health care professionals, and transportation barriers, all of which may impede the successful implementation and sustainability of AI solutions [6-8]. In addition to those challenges, AI solutions also present unique challenges, including black box algorithms [16], privacy issues [19], and limited AI literacy [19,20]. None of the studies focused on any of these user-centered issues. This gap offers rich research opportunities for technology-driven, health-related fields, such as information science and computer science, to address these issues and contribute to a user-centered approach in understanding the development and implementation of AI for health research.

Furthermore, without a qualitative, community-centered approach to AI for health research, which includes understanding the barriers and experiences of rural older adults, the focus may likely remain on clinical outcomes rather than the broader community health outcomes that truly affect these populations [49].

Our literature review revealed a notable absence of initiatives aimed at improving digital and health literacy for rural older adults, highlighting a significant gap in current research. Beyond clinical outcomes, there are valuable opportunities to support the health of rural older adults through efforts focused on enhancing digital literacy as well as education on AI’s impact on health care [19,20]. However, none of the studies within this review investigated older adults’ digital literacy or AI literacy specifically focused on health care tools.

#### Theme 3: Deepening Disparities: Representation of Rurality Missing in AI for Health Research

The findings from this systematic review of the literature highlight a glaring gap in the representation in AI for health research of the perspectives of older adults from rural areas. During the initial screening, we found that using the term “rural” in combination with other key terms frequently yielded no results. As a result, we manually identified references to rural populations within the titles and abstracts. This process underscored not only the lack of focus on rural communities but also the challenges of finding research that includes rural older adults in a meaningful way. AI tools for health care and health decision support are developed using large datasets that, if not inclusive, will continue to marginalize communities like rural older adults [2].

Our review also reveals a geographic imbalance in the literature, with a substantial proportion of studies (11/23, 49%) [23-25,27,33-35,39-41,45] conducted in China. This concentration may suggest a geographic bias in the global development of AI for health research, raising concerns about the applicability and generalizability of findings to rural populations in other countries with different health care systems, cultural contexts, and infrastructure. Moreover, most of the studies we reviewed were heavily urban-centric, emphasizing data from metropolitan populations while overlooking the distinct challenges and health contexts of rural communities. As a result, AI models developed in these contexts may fail to reflect the lived experiences or health care needs of rural older adults, further widening existing disparities [9-11].

Rurality was rarely a central focus in the studies reviewed. In most cases, it was treated as a background demographic variable rather than an analytical lens. Even in studies that addressed rural-related issues, such as health care access or social isolation, the quantitative data often lacked input from rural older adults themselves. This absence of direct perspectives limits the field’s ability to design AI applications that are contextually relevant and responsive to diverse rural settings [13]. Beyond merely including rural populations as a demographic factor, it is essential to deeply understand the lived experiences of rural older adults within AI for health research. In many of the studies reviewed, rurality was treated as a simple comparative statistic, with little exploration of how rural-specific factors might impact research findings or health care outcomes. For example, in many of the studies (15/23, 65%) [24,25,27,29,31-34,36-38,40,42,43,45], rural populations were only mentioned in descriptive statistics, without any further analysis of how rurality influenced the research outcomes. This limited approach prevents a comprehensive understanding of the needs and challenges of rural older adults, ultimately undermining the development of AI solutions and algorithms that are equitable and effective for all populations [17].

### Limitations and Future Directions

Like all systematic literature reviews, this study has limitations. Our search was constrained by the language spoken by the research team, limiting inclusion to studies published in English and potentially omitting relevant research in other languages. In addition, the set of keywords used in the search was not exhaustive, and future reviews using alternative or broader search terms may yield different findings. Despite these limitations, this review serves as an initial step in advancing a research agenda focused on the inclusion of older adults, particularly those from underrepresented, rural populations, in AI for health research. Our primary aim was to identify opportunities where AI can help address unmet health needs and enhance health care delivery for these communities. The findings from this review provide a foundation for the development and implementation of responsible, inclusive AI-driven health care solutions.

### Conclusions

These findings suggest that AI for health care research is currently focused on (1) Numbers over Narrative and (2) Efficacy Over Impact, while (3) Deepening Disparities. While older adults are often described as lagging or in triple jeopardy when it comes to technology in health care, this literature review suggests that it is AI for health research that is actually lagging when it comes to understanding the specific community health care needs of this population. In addition, our findings identify a growing need for increased attention to qualitative research to investigate rural older adults’ perspectives on the role of AI in the development of emerging health care solutions. In addition, there is a need for concerted efforts to examine the impacts of AI for health care research. This includes investigating the transparency of AI tools as well as older adults’ access to education on the implications of AI within their health care.

## Supplementary material

10.2196/70057 Multimedia Appendix 1 Detailed search strategy.

10.2196/70057 Multimedia Appendix 2 Quality assessment for the final sample.

10.2196/70057 Multimedia Appendix 3 Summary of 23 studies in the final sample.

10.2196/70057 Multimedia Appendix 4 Descriptive results (N=23).

10.2196/70057 Checklist 1 Preferred Reporting Items for Systematic Reviews and Meta-Analyses checklist

## Abbreviations

AI: artificial intelligence
JBI: Joanna Briggs Institute
PRISMA: Preferred Reporting Items for Systematic Reviews and Meta-Analyses
