# Supplementary material for: Representation of Rural Older Adults in AI for Health Research: Systematic Literature Review
Source: JMIR Hum Factors. 2025 Sep 15;12:e70057. doi: 10.2196/70057 (PMC12435868; doi:10.2196/70057)
Supplement: Multimedia Appendix 1 [file humanfactors-v12-e70057-s001.pdf]

Round 1: Keyword search

The topic of AI for Health research is an interdisciplinary concern; therefore, we searched a variety of databases across health, multi-disciplinary, and computer science. This search took place in two phases. The medical databases (PubMed, CINAHL Plus with Full Text, PsycINFO) were searched on January 25, 2024. The general and computer science databases (Web of Science, Scopus, ACM DL, iEEE Explore) were searched on March 5th, 2025. All databases were each searched using the following search terms: “artificial intelligence”, “older adults”, “elder”, “senior”, “aging”, “geriatric”, and “rural”. These search terms were informed by the authors’ previous experience with older adults, health literacy, information behavior of minoritized populations, as well as other relevant literature in the field. In our attempt to retrieve recent literature on our topic, we imposed a limit of 10 years of publication, incorporating years between 2013 and 2023. In PubMed, we used built-in limiters including: Full-text, Humans, English (language), Aged: 65+ years and the following search string: (older adults OR elder OR Senior OR Aging OR Geriatric) AND (artificial intelligence) AND (rural). On the same day, we searched CINAHL Plus with Full Text and PsycINFO (through our institutional membership to EBSCOhost) using the same set of keywords as we did in PubMed.

Round 2: Title and abstract screening

In Round 2, titles and abstracts identified in Round 1 were independently screened for inclusion. We predetermined three inclusion and exclusion criteria based on the specific aims of this systematic literature review. We removed articles if they did not meet all three of the inclusion and exclusion criteria. To be included the research needed to (1) focus on AI or include a mention of AI. AI is a broad concept that is inclusive of large language models, machine learning, computer vision, natural language processing, and robotics, all of which enable machines to perform tasks that typically require human intelligence. While other technologies like telehealth and virtual reality are not exclusively considered AI, they can rely on or incorporate algorithms to enhance their outcomes. We selected articles if the term “artificial intelligence” was used to discuss their approach to the study.

Additionally, to be included, the research needed to also (2) focus on older adult participants or include older adult participants’ data and (3) include rural older adult participants. Similar to our approach with the term “artificial intelligence”, the authors recognize that there are multiple and diverging ways of defining rural. For this systematic literature review, we included publications that used the word rural without regard to our own conceptions of rurality. Thus, we included publications that included the word “rural” as a factor of analysis or participant inclusion.

Round 3: Full-text screening

In Round 3, the authors combined their results for full-text screening. In this round, the authors met to discuss their respective results and resolve discrepancies. In this meeting, it was discovered that the results from the CINAHL Plus with Full Text and PsycINFO searches were duplicated within the PUBMED search. Therefore, we removed those results as duplicates.

Round 4: Full-text coding and Full Text Analysis

In Round 4, we deductively coded the final sample of 23 articles for key variables, including publication year, study aim, research method, population characteristics (such as country, sample size, age, and setting), academic fields, application of AI, study objectives, and outcomes. Once the articles were coded, the researchers met to analyze the data using thematic analysis [23]. The main themes identified centered on the representation of older adults in AI for health research.
