# Supplementary material for: Representation of Rural Older Adults in AI for Health Research: Systematic Literature Review
Source: JMIR Hum Factors. 2025 Sep 15;12:e70057. doi: 10.2196/70057 (PMC12435868; doi:10.2196/70057)
Supplement: Multimedia Appendix 2 [file humanfactors-v12-e70057-s002.pdf]

Joanna Briggs Institute's Critical Appraisal Checklist for Analytical Cross-sectional Studies							
Checklist Items	Kim et al., 2023 [27]	Yu et al., 2022 [28]	Lee et al., 2021 [34]	Parry et al., 2023 [39]	Zou et al., 2020 [40]	Liu et al., 2020 [42]	Catalina et al., 2023 [44]
1. Were the criteria for inclusion in the sample clearly defined?	1	1	1	1	1	1	1
2. Were the study subjects and the setting described in detail?	1	1	1	1	1	1	1
3. Was the exposure measured in a valid and reliable way?	1	1	1	1	1	1	1
4. Were objective, standard criteria used for measurement of the condition?	1	1	1	1	1	1	1
5. Were confounding factors identified?	1	1	0	1	1	0	1
6. Were strategies to deal with confounding factors stated?	1	1	0	1	1	0	1
7. Were the outcomes measured in a valid and reliable way?	1	1	1	1	1	1	1
8. Was appropriate statistical analysis used?	1	1	1	1	1	1	1
**Rating**	**100% (excellent)**	**100% (excellent)**	**75% (good)**	**100% (excellent)**	**100% (excellent)**	**75% (good)**	**100% (excellent)**

Joanna Briggs Institute's Critical Appraisal Checklist for Qualitative Studies			
Checklist Items	Cherry et al., 2017 [29]	Lee et al., 2017 [32]	Randall et al., 2018 [33]
1. Is there congruity between the stated philosophical perspective and the research methodology?	1	1	1
2. Is there congruity between the research methodology and the research question or objectives?	1	1	1
3. Is there congruity between the research methodology and the methods used to collect data?	1	1	1
4. Is there congruity between the research methodology and the representation and analysis of data?	1	1	1
5. Is there congruity between the research methodology and the interpretation of results?	1	1	1
6. Is there a statement locating the researcher culturally or theoretically?	0	0	1
7. Is the influence of the researcher on the research, and vice- versa, addressed?	0	0	1
8. Are participants, and their voices, adequately represented?	1	1	1
9. Is the research ethical according to current criteria or, for recent studies, and is there evidence of ethical approval by an appropriate body?	1	1	0
10. Do the conclusions drawn in the research report flow from the analysis, or interpretation, of the data?	1	1	0
**Rating**	**80% (good)**	**80% (good)**	**80% (good)**

Joanna Briggs Institute's Critical Appraisal Checklist for Cohort Studies				
Checklist Items	Wu et al., 2022 [26]	Yi et al., 2022 [37]	Manemann et al., 2021 [43]	Zhang et al., 2022 [46]
1. Were the two groups similar and recruited from the same population?	1	1	1	1
2. Were the exposures measured similarly to assign people to both exposed and unexposed groups?	1	1	1	1
3. Was the exposure measured in a valid and reliable way?	1	1	1	1
4. Were confounding factors identified?	1	1	0	1
5. Were strategies to deal with confounding factors stated?	1	1	0	1
6. Were the groups/participants free of the outcome at the start of the study (or at the moment of exposure)?	1	1	1	1
7. Were the outcomes measured in a valid and reliable way?	1	1	1	1
8. Was the follow up time reported and sufficient to be long enough for outcomes to occur?	0	1	1	1
9. Was follow up complete, and if not, were the reasons to loss to follow up described and explored?	1	1	0	1
10. Were strategies to address incomplete follow up utilized?	1	1	0	1
11. Was appropriate statistical analysis used?	1	1	1	1
**Rating**	**91% (excellent)**	**100% (excellent)**	**64% (fair)**	**100% (excellent)**

Joanna Briggs Institute's Critical Appraisal Checklist for Quasi-experimental Studies			
Checklist Items	Jin et al., 2023 [30]	Zhu et al., 2022 [36]	Jones et al., 2021 [45]
1. Is it clear in the study what is the “cause” and what is the “effect” (i.e. there is no confusion about which variable comes first)?	1	1	1
2. Was there a control group?	0	0	0
3. Were participants included in any comparisons similar?	1	0	1
4. Were the participants included in any comparisons receiving similar treatment/care, other than the exposure or intervention of interest?	1	0	1
5. Were there multiple measurements of the outcome, both pre and post the intervention/exposure?	1	1	1
6. Were the outcomes of participants included in any comparisons measured in the same way?	1	1	1
7. Were outcomes measured in a reliable way?	1	1	1
8. Was follow-up complete and if not, were differences between groups in terms of their follow-up adequately described and analyzed?	1	0	1
9. Was appropriate statistical analysis used?	1	1	1
**Rating**	**89% (good)**	**56% (fair)**	**89% (good)**

Joanna Briggs Institute's Critical Appraisal Checklist for Randomized Controlled Trials		
Checklist Items	Boman et al., 2014 [31]	Mathenge et al., 2022 [38]
1. Was true randomization used for assignment of participants to treatment groups?	1	1
2. Was allocation to treatment groups concealed?	0	1
3. Were treatment groups similar at the baseline?	1	1
4. Were participants blind to treatment assignment?	0	0
5. Were those delivering the treatment blind to treatment assignment?	0	1
6. Were treatment groups treated identically other than the intervention of interest?	1	1
7. Were outcome assessors blind to treatment assignment?	0	1
8. Were outcomes measured in the same way for treatment groups?	1	1
9. Were outcomes measured in a reliable way?	1	1
10. Was follow up complete and if not, were differences between groups in terms of their follow up adequately described and analysed?	1	1
11. Were participants analysed in the groups to which they were randomized?	1	1
12. Was appropriate statistical analysis used?	1	1
13. Was the trial design appropriate and any deviations from the standard RCT design (individual randomization, parallel groups) accounted for in the conduct and analysis of the trial?	1	1
**Rating**	**69% (fair)**	**92% (excellent)**

Joanna Briggs Institute's Critical Appraisal Checklist for Diagnostic Test Accuracy Studies				
Checklist Items	Cui et al., 2023 [24]	Su et al., 2021 [25]	Ding et al., 2023 [35]	Tang et al., 2018 [41]
1. Was a consecutive or random sample of patients enrolled?	1	0	1	1
2. Was a case control design avoided?	1	1	1	1
3. Did the study avoid inappropriate exclusions?	1	1	1	1
4. Were the index test results interpreted without knowledge of the results of the reference standard?	1	1	1	1
5. If a threshold was used, was it pre-specified?	1	1	1	1
6. Is the reference standard likely to correctly classify the target condition?	1	1	1	1
7. Were the reference standard results interpreted without knowledge of the results of the index test?	1	1	1	1
8. Was there an appropriate interval between index test and reference standard?	1	1	1	1
9. Did all patients receive the same reference standard?	1	1	1	1
10. Were all patients included in the analysis?	1	0	0	0
**Rating**	**100% (excellent)**	**80% (good)**	**90% (good)**	**90% (good)**
