# Supplementary material for: Representation of Rural Older Adults in AI for Health Research: Systematic Literature Review
Source: JMIR Hum Factors. 2025 Sep 15;12:e70057. doi: 10.2196/70057 (PMC12435868; doi:10.2196/70057)
Supplement: Multimedia Appendix 3 [file humanfactors-v12-e70057-s003.pdf]

Authors	Field	Year	Country	Study Aims	Methods	Years of "Older Adult" Inclusion	Total Sample Size (N)	Older Adult Sample Size (n)	Rural Context	Outcomes
Cui T, et al. [24]	Health Science	2023	China	Investigate the use of deep learning systems in screening images for retinal lesions in rural older adult patients.	Quantitative	50 and older	N=3149		24 Villages of Yangxi County, China.	Deep learning systems performed well as a retinal lesion screening tool for rural populations.
Su D, et al. [25]	Psychology	2021	China	Examine the application of machine learning models in the predicting the risk of depression in older adults.	Quantitative	65 and older	N=1538	n=644	Rural not a focus, but identified.	Machine learning models based on long short-term memory technology were found to be beneficial in identifying predictors for elderly with depression.
Wu Y, et al. [26]	Psychology	2022	China	Explore the use of machine learning to predict long-term cognitive trajectories.	Quantitative	65 and older	N=3502	n=1850	Rural not a focus, but discussed	Machine learning algorithms demonstrated potential for classifying trajectories which would aid in early cognitive function risk identification.
Kim J, et al. [27]	Health Science	2023	South Korea	Use machine learning to develop a predictive model for identifying suicidal ideation risk for rural older adults.	Quantitative	65 and older	N=650		Rural South Korea	Machine learning predictive models were found to successfully predict the risk of suicidal ideation in older adults in rural populations.
Yu J, et al. [28]	Gerontology	2022	China	Use machine learning to investigate factors of active aging among older adults.	Quantitative	60 and older	N=7503	n=4561	Rural and Urban China	Machine learning models identified of determinants of active aging including health, residency (urban/rural), neighborhood infrastructure, social activities, and living with family members.
Cherry C, et al. [29]	Rehabilitation	2017	United States	Determine participants' experiences of robotic therapy devices used for telerehabilitation.	Qualitative	50 and older	N=10		Rural Veterans in 2 districts in the state of Georgia	Patients reported their positive experiences with telerehabilitation, suggesting that telerehabilitation robots can be effective in extending effective rehabilitations services to rural Veterans with limited access to care.
Jin H, et al. [30]	Neuroscience	2023	India	Use of machine learning to estimate the prevalence of dementia in older adults in India.	Quantitative	60 and older	N=31,477	n=4096	Rural not a focus, but discussed.	Semi-supervised machine learning approaches can help epidemiological aging studies identify prevalence of dementia and provide information for long term public planning and social support.
Boman K, et al. [31]	Health Science	2014	Sweden	Evaluate the feasibility of robot-assisted, remote echocardiography consultation.	Quantitative	55 and older	N=38		Rural primary healthcare center in Sweden.	Robot-assisted echocardiography consultation reduced time to attaining a specialist consultation which advances telemedicine practices.
Lee H, et al., [32]	Psychology	2017	United States	Design socially assistive robots with older adults diagnosed with depression and their therapists, and identify challenges.	Qualitative	Did not specify	N=15	n=5	Rural not a focus, but discussed as limitation.	The participatory design (PD) methodology engaged older adults with depression and their therapists in co-designing socially assistive robots. The approach contributed valuable insights for creating more effective and context-sensitive assistive technologies.
Randall N, et al., [33]	Health Science	2018	United States	Engage older adults as co-designers.	Qualitative	55 and older	N=19	n=9	Rural not a focus, but discussed.	Participants did not fully assume the role of co-designer at the conclusion of the study. However, they did become more engaged with the process as time progressed.
Lee C, et al., [34]	Health Science	2021	China	Investigate and analyze the demand for smart healthcare services for the elderly. Investigate and analyze the current situation of supply accessibility for smart healthcare service for the elderly.	Qualitative	60 and older	N=948		Rural not a focus, but discussed.	Older adult participants have a low level of understanding of smart healthcare services. And, 81.6% of older adults have never purchased /leased smart healthcare products by themselves.
Ding Z, et al., [35]	Neuroscience	2023	China	Develop a simplified and visualized score for rural stroke risk assessment, which is combined with a machine learning (ML) algorithm.	Quantitative	55 and older	N = 38,322		Rural not a focus, but identified.	Developed a convenient tool for stroke risk assessment among rural residents, which is valuable for identifying individuals with higher stroke risk.
Zhu T, et al., [36]	Health Science	2022	China	Design and develop a service management system, which introduces Internet of things technology into intelligent elderly care services to improve the efficiency of elderly care services.	Quantitative	Did not specify (used participants from elderly care)	N=50		Rural villages and towns in Beijing	Result sshow that the system can strengthen the sharing of elderly care information, reduce the workload of service personnel to a certain extent, and improve the efficiency and quality of service

Yi, SE, et al., [37]	Health Science	2022	Canada	Develop and internally validate a single, large- scale, machine learning model to predict hospitalizations due to ACSCs in a cohort of 1.85 million older adults in Ontario, Canada.	Quantitative	65 and older	N=1,854,116		Rural not a focus, but identified.	Demonstrated that the development and validation of a single, large-scale machine learning model to predict the 1-year risk of hospitalization from a series of ambulatory-care sensitive conditions is feasible in a large and diverse cohort of seniors using AHD.
Mathenge W, et al., [38]	Health Science	2022	Rwanda	Assess whether use of Orbis International's Cybersight AI in diabetes clinics leads to increased patient uptake of DR referral services.	Quantitative	55 and older	N =275		Rural not a focus, but identified.	Demonstrated the potential of AI-supported DR screening to deliver increased uptake of referral services.
Parry, M, et al., [39]	Health Science	2023	United States	Characterize the general patient opinion and identify factors that influence these opinions concerning the implementation of AI/ML in orthopaedic surgery across multiple academic and community health centers	Quantitative	55 and older	N = 397	n=237	Rural not a focus, but identified.	Patients appeared comfortable with the use of AI in their care. A patient's overall comfort level appeared to be influenced by age, education level, knowledge of AI, and perceptions of the effects of the technology on clinical outcomes and healthcare costs. Patients do not appear comfortable with autonomous AI-driven surgical robots.
Zou, FW, et al., [40]	Health Science	2020	China	Assess the concordance between Watson for Oncology's (WFO) treatment recommendations and real-world clinical decisions made for cervical cancer patients. Determine WFO's suitability as a decision-support tool for cervical cancer treatment in the Chinese health-care context.	Quantitative	65 and older	N=300	n=52	Rural and Urban China	WFO could be an effective decision-support tool in cancer therapy for Chinese physicians, it also helps to standardize the treatment of cervical cancer.
Tang H, et al., [41]	Health Science	2018	China	Use machine learning methods to analyze lung cancer related risk factors, and try to find a way to predict it, based on research of medical follow-up data.	Quantitative	50 and older	N=510,000		Rural not a focus, but discussed.	The key outcome is that the best-performing model—leveraging classification methods—achieved a high accuracy rate, indicating promising potential for early lung cancer detection
Liu F, et al., [42]	Gerontology	2020	China	Analyze the impacts of different education level on the elderly care mode selection and the demands for elderly care services based on the questionnaire survey for under and the group of 60 years old and over.	Quantitative	60 and older	N=649		Rural home-based care in China.	The study concludes that an elderly person's education level plays a crucial role in their selection of care modes and eye toward advanced care services, indicating a need for the elderly-care sector to tailor services to these demographic nuances.
Manemann, S, et al., [43]	Health Science	2021	United States	Capitalize on the depth and breadth of clinical data within the HER (electronic health record) systems to revolutionize risk prediction and to optimize personalized care for every patient.	Quantitative	Did not specify (included adults 30 and over).	N=409,715		Rural not a focus, but identified.	Establishes comprehensive EHR-based cohorts and processes vast quantities of data to support the development of predictive models for complex disease through advanced machine learning methods.
Catalina, QM, et al., [44]	Health Science	2023	Germany	Describe the population's perception and knowledge of the use of AI in radiology. To identify possible sociodemographic factors related to develop strategies to increase acceptance and confidence in AI.	Quantitative	65 and older	N = 379	n=53	Rural not a focus, but identified.	The majority of the study population reported being familiar with the concept of AI, with varying degrees of acceptance of its implementation in radiology.
Jones, KJ, et al., [45]	Health Science	2021	United States	Evaluate the effectiveness of using automated video monitoring systems (AVMS) to decrease the risk of unattended bed exits (UBE) among patients at high risk for falls and fall-related injuries in 15 small rural hospitals.	Quantitative	65 and older	N=358	n=329	Small, rural hospitals in the U.S.	The implementation of AVMSs in small rural hospitals was associated with significant reductions in UBEs and fall-related injuries among high-risk patients. The system's high sensitivity and reasonable predictive value suggest that AVMSs can be an effective tool for enhancing patient safety in such settings.
Zhang, L, et al., [46]	Health Science	2022	China	Develop and validate a simple, efficient, and joint machine learning model for identifying individuals at high risk of CHD using easily obtainable nonlaboratory parameters.	Quantitative	65 and older	N=38,716		Rural not a focus, but identified.	The machine learning-based model comprising readily accessible variables accurately identified individuals at high risk of CHD.
