# Supplementary figures and images for: Representation of Rural Older Adults in AI for Health Research: Systematic Literature Review
Source: JMIR Hum Factors. 2025 Sep 15;12:e70057. doi: 10.2196/70057 (PMC12435868; doi:10.2196/70057)

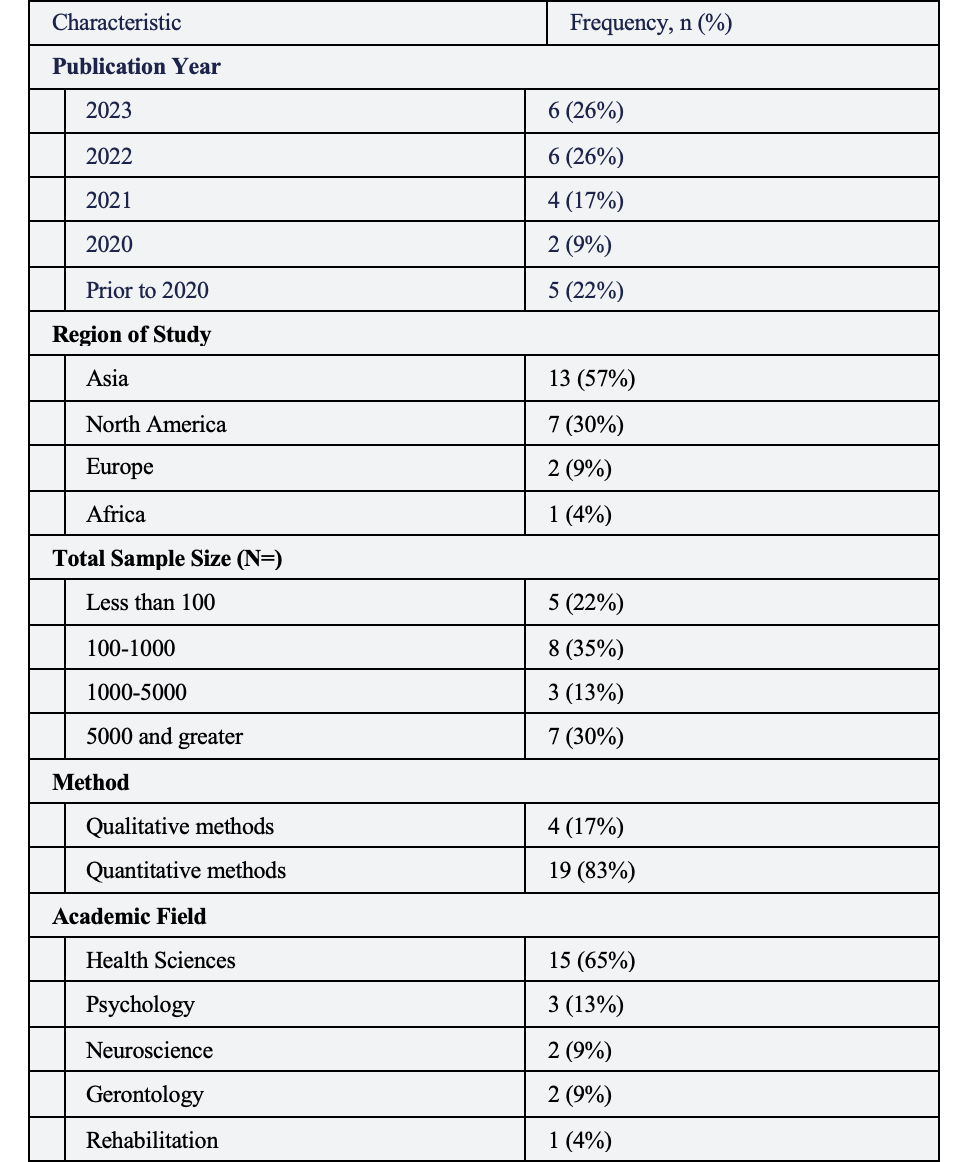

Supplement: Multimedia Appendix 4 [file humanfactors-v12-e70057-s004.png]
